# Efficient *Agrobacterium*‐Mediated Transformation of Green *Arabidopsis* Suspension Cells

**DOI:** 10.1002/biot.70145

**Published:** 2025-10-24

**Authors:** Matthias Buntru, Stefano Di Fiore, Nils Hahnengress, Helga Schinkel, Stefan Schillberg, Greta Nölke

**Affiliations:** ^1^ Fraunhofer Institute for Molecular Biology and Applied Ecology Aachen Germany; ^2^ Institute for Molecular Biotechnology RWTH Aachen University Aachen Germany

## Abstract

Photosynthetic plant cell suspension cultures are a valuable experimental system for analyzing various physiological processes. This system bypasses the structural complexity of the whole plant organism and can be manipulated under uniform conditions. However, achieving a highly efficient and consistent transformation of plant suspension cells remains challenging. By using green fluorescent protein (GFP) and a microplate confocal imaging system for high‐throughput analysis, we optimized the transformation of green *Arabidopsis* suspension cells to infect almost 100% of the cells. Key elements of our protocol included using the hypervirulent *Agrobacterium tumefaciens* strain AGL1, co‐cultivating agrobacteria and suspension cells on solidified medium plates, and adding AB minimal salts and the surfactant Pluronic F68. The presented method can significantly increase the transformation rate of plant suspension cells, facilitating the introduction of genetic pathways for producing industrial, cosmetic, or pharmaceutical compounds in these systems.

## Graphical Abstract and Lay Summary

We present a highly efficient and reproducible *Agrobacterium*‐mediated transformation protocol for photosynthetic *Arabidopsis* suspension cells, achieving infection rates of almost 100% within 5 days.

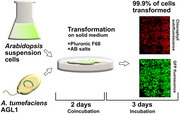

## 1 Introduction

*Agrobacterium*‐mediated plant transformation is a widely used technique for introducing foreign genes into plant cells. It is based on the natural ability of *Agrobacterium tumefaciens*, a bacterium found in soil, to transfer a part of its DNA (known as T‐DNA) into the plant genome. This technique has several advantages over other methods of plant transformation, including low cost, high efficiency, and stable integration, as well as the ability to transfer large DNA fragments [1]. It can be used for both stable and transient expression of transgenes [2] and for genome editing using engineered nucleases such as CRISPR/Cas9 [3] and TALENs [4]. This method has been employed to introduce foreign genes into plant cells for various applications, such as analyzing gene functions [5] or determining protein localization [6]. However, low transformation efficiencies often limit its use in high‐throughput applications. Factors affecting the efficiency of DNA transfer from *A. tumefaciens* to plant cells have been identified and range from *Agrobacterium* strain and vector design to co‐cultivation conditions such as temperature and duration. The co‐cultivation medium, that is, its pH and the addition of chemicals and/or surfactants, can also influence transformation efficiency [7].

Although many studies on *Agrobacterium*‐mediated transformation of plants have been conducted, protocols for the transformation of plant suspension cells and calli are much rarer. Such protocols are mostly employed to produce stably transformed plant suspension culture lines, for which transformation efficiency is of minor importance [8, 9]. The previous focus of optimizing transformation methods for in vitro cultured plant cells was mainly on refining established approaches by adjusting parameters such as the bacterial strain [6], bacterial concentration, inoculation time, and co‐cultivation period [10]. Furthermore, the transformation efficiency of in vitro‐cultured plant cells can be increased through co‐cultivation under continuous light [11] or on a solid medium [12]. However, transient transformation is a valuable tool for quickly gaining insight into construct functionality or for speedily producing small amounts of recombinant proteins. If the transformation efficiency is high enough, clear results can be obtained within a week, and high‐throughput scenarios can be realized in microtiter plates. Heterotrophic white suspension cells are also nearly always the object of these protocols, as the number of established photoautotrophic or photomixotrophic plant suspension cultures is limited [13]. However, for studying photosynthesis, source‐sink regulation, or producing certain secondary metabolites, a photosynthetically active suspension culture is necessary [14, 15], as is an effective procedure for transforming such green suspension cells.

In this article, we investigated the effectiveness of various factors in developing a highly efficient method for *Agrobacterium*‐mediated transformation of photosynthetically active *Arabidopsis* suspension cells. Using a microplate confocal imaging system, we employed a novel, image‐based, high‐throughput method to analyze transformation efficiency. The resulting transformation protocol has the potential to significantly improve the efficiency with which plant suspension cells can be transformed.

## 2 Materials and Methods

### 2.1 Plant Material, Agrobacteria, and Plasmid

Green *Arabidopsis thaliana* cv. Columbia suspension cells were cultivated in a Murashige and Skoog medium MS1: MS medium including vitamins (M0222, Duchefa Biochemie, Haarlem, The Netherlands) supplemented with 5 g/L sucrose, 0.25 mg/L 6‐benzylaminopurine (BAP), and 0.5 mg/L 2,4‐dichlorophenoxyacetic acid (2,4‐D), at a pH of 5.0 [13]. The cells were cultivated in a shaker at 80 rpm and 24°C under a 16/8 h light/dark photoperiod with an irradiance of 110 µmol photons m^−2^ s^−1^ at the culturing surface. The medium was renewed every week by transferring 10% (v/v) of the packed cell volume (PCV) into 100 mL of fresh medium.

Electrocompetent *A. tumefaciens* strain AGL1 [16] (product no. 1283‐12, Intact Genomics, St. Louis, MO 63146, USA) was used.

A double‐stranded DNA fragment encoding enhanced green fluorescent protein (GFP) (GenBank: AAB02572.1) with a C‐terminal streptavidin affinity tag was synthesized (Integrated DNA Technologies, Coralville, IA, USA), including overhangs suitable for Golden Gate Assembly [17]. This DNA fragment was introduced into pICH86988 (MoClo Toolkit, Addgene, Watertown, MA 02472, USA) at BsaI sites using Golden Gate Assembly. The vector was checked for the correct sequence and then multiplied according to standard procedures.

### 2.2 *Agrobacterium* Transformation

*A. tumefaciens* was transformed by electroporation and selected on LB agar plates supplemented with 50 µg/mL of carbenicillin and 25 µg/mL of kanamycin. Colonies typically emerged following a 3‐day incubation period at 28°C. Transformation was verified via check‐PCR, after which glycerol stocks were prepared.

### 2.3 Transformation of Plant Cells by *Agrobacterium*

An *Agrobacterium* preculture was inoculated from a glycerol stock in YEB+ medium (5 g/L beef extract, 1 g/L yeast extract, 5 g/L peptone from casein, 5 g/L sucrose, and 2 mM MgSO_4_) containing 50 µg/mL carbenicillin and 25 µg/mL kanamycin. After cultivation (28°C; 160 rpm) for 20–24 h, the preculture was used to inoculate AB‐MES medium (17.2 mM K_2_HPO_4_, 8.3 mM NaH_2_PO_4_, 18.7 mM NH_4_Cl, 2 mM KCl, 1.25 mM MgSO_4_, 100 µM CaCl_2_, 10 µM FeSO_4_, 50 mM MES, and 20 g/L glucose, pH 5.5) [18] containing appropriate antibiotics and 200 µM acetosyringone as a main culture (OD_600_ = 0.2). The main culture was incubated at 28°C and 160 rpm for 16–20 h, after which 50 mL of the *Agrobacterium* culture (OD_600_ = 0.3–0.5) was harvested by centrifugation at 6800 ×  *g* for 10 min. The cells were then resuspended in ABM‐MS medium (50% [v/v] AB‐MES, 1.1 g/L MS basal salts [M0221, Duchefa Biochemie], 0.25% [w/v] sucrose, pH 5.5) [5] to an OD_600_ of 0.8.

An *Arabidopsis* suspension culture was initiated with a PCV of 10% and grown for 4−5 days to the mid‐exponential phase, typically yielding a PCV of 15%−20%.

Co‐cultivation was performed in four different ways using two different liquid media and two different solid media.

For co‐cultivation in a liquid medium, the *Arabidopsis* suspension culture was diluted 1:4 in either MS1 medium or ABM‐MS medium [5] immediately prior to being cultured with agrobacteria. Three milliliters of the diluted *Arabidopsis* suspension culture and 50 µL of the *Agrobacterium* suspension (OD_600_ = 0.8) were mixed in a six‐well cell culture plate. Acetosyringone to a final concentration of 200 µM was added, the plate was sealed with Micropore tape and was incubated at 24°C and 160 rpm under continuous light (∼90 PPFD) for 2 days.

For co‐cultivation on a solid medium, the *Arabidopsis* suspension cells were washed twice (centrifugation at 200 × *g* for 5 min; supernatant discarded) using ABM‐MS medium. After the second wash, the PCV was adjusted to 70% (v/v) using ABM‐MS medium. One milliliter of the *Agrobacterium* suspension (OD_600_ = 0.8) was centrifuged in a 2‐mL centrifuge tube (5000 × *g*, 5 min). The pellet was resuspended in 30 µL of ABM‐MS medium and mixed with 1 mL of the washed *Arabidopsis* cells and 200 µM acetosyringone. Then, 0.5 mL of the suspension was dropped onto a Petri dish containing either solid Paul's medium (4.3 g/L MS basal salts (M0221, Duchefa Biochemie), 10 g/L sucrose, and 8 g/L plant agar, pH 5.8) [12] or solid ABM‐MS medium containing 8 g/L plant agar. The plate was rotated by hand to spread the cells, after which excess liquid was allowed to dry off under the clean bench for 10 min before sealing with Micropore tape. The plate was then incubated at 24°C under continuous light (∼90 PPFD) for 2 days.

The four co‐cultivations described above were also performed with 0.05% (w/v) Pluronic F68 (PF68) in the liquid or on the solid medium [8]. As a control, *Arabidopsis* suspension cells without added agrobacteria were incubated in the same way as the cells in the four different co‐culture setups. Before analysis (after 2 days), the cells were carefully removed from the six‐well plates using cut pipette tips (liquid) or a spatula and liquid ABM‐MS medium (solid). The cells were then washed twice with ABM‐MS medium containing 250 µg/mL ticarcillin to reduce the concentration of *Agrobacterium*. To regenerate, the washed, co‐cultivated cells were dropped (100 µL/well) on solid MS1 medium (containing 8 g/L plant agar) supplemented with 250 µg/mL ticarcillin and 50 µg/mL kanamycin in a 12‐well cell culture plate (product no. 665970, Greiner Bio‐One, Frickenhausen, Germany) and incubated for 3 days at 24°C under continuous light (∼90 PPFD).

### 2.4 Microscopy and Image Analysis

To analyze the transformation efficiency of *Arabidopsis* cells after 2 days, the cells were diluted 1:10 with ABM‐MS medium, plated in black 96‐well half area plates (product no. 675096, Greiner Bio‐One), and left to sediment for 10 min prior to imaging. Analysis after three additional days incubation was done directly in transparent 12‐well plates through the agar layer. Images were acquired using an Opera QEHS 2.0 automated high‐content screening system (PerkinElmer, Rodgau, Germany) with an overall magnification of 40×. For cells diluted in liquid growth medium in 96‐well plates, 5–6 images were acquired to cover almost the entire well. For cells grown in 12‐well plates, 15 images per well were acquired at random positions. We imaged the cells by autofluorescence of chlorophyll, using a 561 nm laser for excitation in combination with a 690/50 nm bandpass emission filter. To image GFP‐positive cells, we used a 488 nm laser for excitation and a 540/75 nm emission filter. To prevent cross‐talk between the GFP and chlorophyll signals, the microscope was set to automatically acquire the chlorophyll and GFP channels consecutively. Image analysis was performed using Acapella 2.0 software (PerkinElmer) and a custom‐made script. The script identified cells in the chlorophyll image and generated a mask comprising the border of each cell, which was then superimposed onto the GFP image. This produced several data for both channels, such as the number of cells per well, the median and average intensity of each cell. These data were analyzed in Excel as follows: throughout the experiment, we included control wells containing untreated wild‐type (WT) *Arabidopsis* cells whose median fluorescence intensity in the GFP channel was used to set a 99.5th percentile‐based intensity threshold. This threshold excluded false positive signals due to the WT cells’ intrinsic background autofluorescence. Transformed cells that scored higher GFP intensity than the WT cells’ threshold were classified as GFP‐positive. The transformation efficiency in percent was calculated for each of the conditions tested by dividing the number of GFP‐positive cells by the total number of cells detected in the chlorophyll channel.

### 2.5 Statistical Analysis

Student's *t*‐tests were performed to analyze the statistical significance between the experimental groups.

## 3 Results

After 2 days of co‐cultivation, the GFP fluorescence of the *Arabidopsis* suspension cells was analyzed as evidence of transformation. On average, 3215 cells (±921 standard deviation) were analyzed in one well for co‐cultivation in liquid medium, and 4641 cells (±1531 standard deviation) were analyzed for co‐cultivation on solid medium. The numbers differ slightly due to the preparation procedure of the *Arabidopsis* cells; however, this difference is not significant (Student's *t*‐test: *p* = 0.16), and the numbers are consistent with those of the control wells without agrobacteria (average: liquid 3797 cells; solid 4455 cells).

No cells with GFP‐fluorescence were detected in any of the control wells, while all co‐cultivation setups produced transformed cells (i.e., cells showing GFP‐fluorescence), albeit with varying efficiencies (see Table 1). The transformation efficiency ranked as follows: solid Paul's medium (1.8%) < liquid MS1 medium (2.4%) < liquid ABM‐MS medium (10.7%) < solid ABM‐MS medium (13.6%), which achieved the best result. Each medium was also used with the addition of 0.05% (w/v) PF68 surfactant, which had a slightly positive effect on the transformation efficiency of both liquid media (MS1: +1.4%; ABM‐MS: +0.9%), while the transformation efficiency decreased to a similar extent with the use of PF68 on solid media (Paul's: −1%; ABM‐MS: −1.5%).

**TABLE 1:** Transformation efficiency (%) evaluated after 2 and 5 days by automated microscopy and image analysis.

		Co‐cultivation (2 days)	Co‐cultivation + 3 days incubation (5 days)
**Liquid media**	MS1	2.4	n.a.
	MS1 + PF68	3.8	n.a.
	ABM‐MS	10.7	54.9
	ABM‐MS + PF68	11.6	76.2
**Solid media**	Paul's	1.8	n.a.
	Paul's + PF68	0.8	n.a.
	ABM‐MS	13.6	99.4
	ABM‐MS + PF68	12.1	99.9

*Note*: Each experiment also included an evaluation of non‐transformed WT cells that were treated in the same way. These controls did not exhibit any green fluorescence.

Abbreviation: n.a. = not analyzed.

**FIGURE 1:**
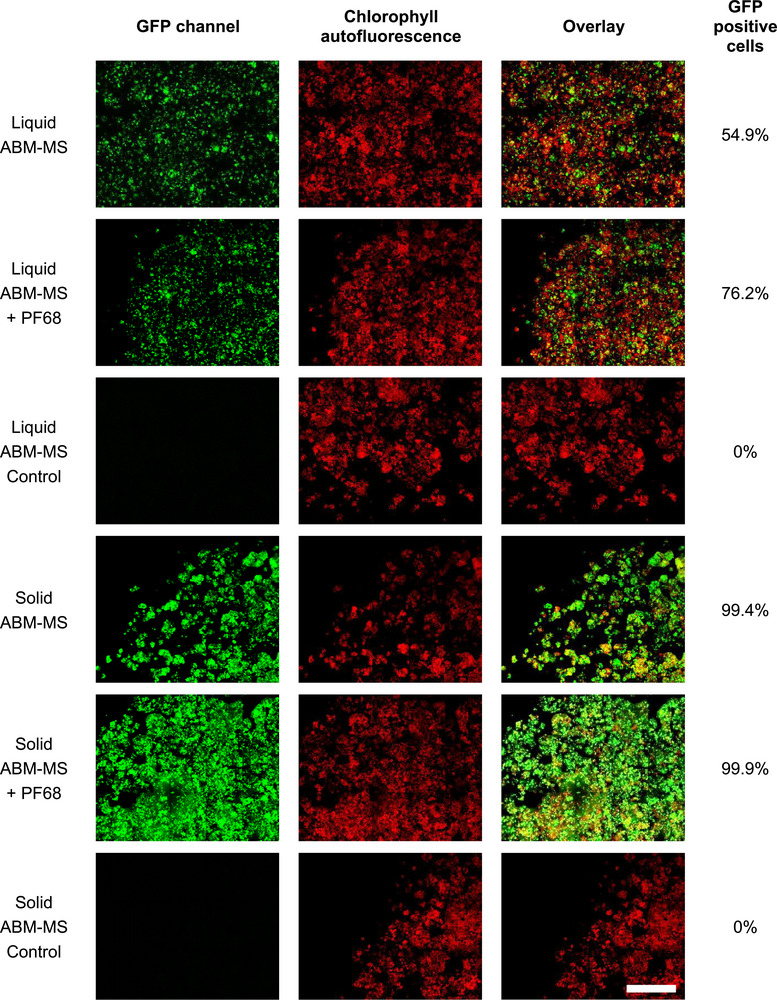
Analysis of GFP‐positive *Arabidopsis* cells after co‐cultivation with *Agrobacterium* in liquid or on solid ABM‐MS medium for 2 days with or without 0.05% (w/v) PF68 and incubation on solid medium for 3 days. Images were then acquired with a microplate confocal imaging system. “Control” are cells that were incubated without *Agrobacterium*. The column on the right of the figures shows the percentage of GFP‐positive cells, as calculated by image analysis and data processing. Scale bar = 2000 µm.

Assuming that not all *Arabidopsis* cells expressed GFP directly after 2 days of co‐cultivation, cells from co‐cultivations with the highest transformation rate in liquid and on solid ABM‐MS with and without PF68 as well as control cells were transferred to solid MS1 medium in a 12‐well cell culture plate and analyzed again 3 days later. On average, 15,387 cells were analyzed (± 4494 standard deviation). Again, no GFP fluorescence was detected in the control wells. Transformation efficiencies rose to 54.9% (liquid co‐cultivation) and 99.4% (solid co‐cultivation). The efficiency was considerably higher for liquid co‐cultivation with PF68 in the medium (76.2%) and minimally increased for solid co‐cultivation (99.9%) (Table 1, Figure 1).

## 4 Discussion

The transformation of plant suspension cells with *A. tumefaciens* is a method that has been used for many years [19]. Using a hypervirulent *A. tumefaciens* strain such as AGL1 is recommended, as it has transformed green *Arabidopsis* suspension cells in all the experiments presented here. Due to stronger expression of the *vir* genes, AGL1 is preferred for recalcitrant plant cells. Furthermore, the *Agrobacterium* strain selected must be matched to the plant being transformed [20], so different strains may be better suited to transforming different plant suspension cells.

The best result—a transformation rate approaching 100%—was achieved by adding AB minimal salts [5] and using a solid co‐cultivation medium [12]. AB‐based media are optimal for *vir* gene induction [5]. Furthermore, the transfer of the T‐DNA requires physical contact between bacterial and plant cells, established by a protein structure called the T‐pilus. This structure is similar to the conjugational bridge formed between mating bacteria [21]. The increased efficiency of DNA transfer when cells are plated on a solid medium has previously been observed for conjugation between different *Agrobacterium* strains [22], as well as for the transformation of tobacco BY‐2 cells [12].

When used in solid co‐cultivation medium, the addition of surfactant PF68 had no significant impact on transformation efficiency. However, an approximately 20% higher transformation efficiency was observed after a 5‐day incubation period in a liquid co‐cultivation medium, which is consistent with the results obtained for the transformation of suspension cells of *Artemisia pallens* [8]. Surfactants such as PF68 can improve T‐DNA delivery by helping *Agrobacterium* to attach to plant cells [7].

The continuous light during co‐cultivation may have contributed to the high transformation efficiency observed. Zambre et al. [11] proposed that light generally promotes *Agrobacterium*‐mediated T‐DNA transfer to plant cells. It is most likely that light influences T‐DNA transfer frequencies not through the bacteria, but through the competence of plant cells for *Agrobacterium* attachment or T‐DNA uptake. Light may change physiological factors that influence this ability, such as plant hormone levels, cell growth, and cell cycle stage [23, 24]. However, the exact reason for the light's stimulating effect remains to be determined. As the experiments were performed using a photomixotrophic suspension culture cultivated in light, it was logical to maintain a similar regime during co‐cultivation.

To analyze the effects of different co‐cultivation conditions, we established an automated microscopy method using red chlorophyll autofluorescence to determine total cell numbers [25] and green fluorescence of GFP to determine the percentage of successfully transformed cells, similar to the protocol used by Page et al. [26] for rice protoplasts. After 2 days of co‐cultivation, transformed cells could be detected; however, the full extent of transformation was not apparent until after an additional 3 days of incubation. By using automated high‐content screening and image analysis, we show that this method is ideal for high‐throughput experiments to easily identify optimal transformation conditions for *Arabidopsis* suspension cells. However, although not yet tested, it is highly likely that this transformation protocol is not restricted to *Arabidopsis* cells but also applicable to suspension cells of other species or perhaps even thin leaves, making it a valuable tool for plant molecular biology studies.

## 5 Conclusion

This study presents a highly efficient protocol for the transformation of green *Arabidopsis* suspension cells using *Agrobacterium*, resulting in an almost 100% success rate. The critical factors were the use of the hypervirulent AGL1 *Agrobacterium* strain and co‐cultivation on a solid medium containing AB salts.

## Author Contributions

MB conceived and designed the study, performed experiments, curated and validated data, and wrote the original draft of the manuscript. SDF contributed to methodology development, data visualization, validation, and formal analysis, and participated in writing and revising the manuscript. NH performed experiments and prepared visualizations. HS contributed to manuscript writing and critical revision. SS contributed to manuscript review and provided funding. GN supervised the project, acquired funding, and contributed to project administration and manuscript revision.

## Conflicts of Interest

The authors declare no conflicts of interest.

## Data Availability

The data that support the findings of this study are available from the corresponding author upon reasonable request.
